# Concerns About Information Regarding COVID-19 on the Internet: Cross-Sectional Study

**DOI:** 10.2196/20487

**Published:** 2020-11-09

**Authors:** Yusui Zhao, Shuiyang Xu, Lei Wang, Yu Huang, Yue Xu, Yan Xu, Qiaohong Lv, Zhen Wang, Qingqing Wu

**Affiliations:** 1 Zhejiang Provincial Center for Disease Control and Prevention Hangzhou China

**Keywords:** coronavirus, COVID-19, disease prevention, internet, knowledge

## Abstract

**Background:**

Since the outbreak of COVID-19, the Chinese government and the Chinese Center for Disease Control and Prevention have released COVID-19–related information to the public through various channels to raise their concern level of the pandemic, increase their knowledge of disease prevention, and ensure the uptake of proper preventive practices.

**Objective:**

Our objectives were to determine Chinese netizens’ concerns related to COVID-19 and the relationship between their concerns and information on the internet. We also aimed to elucidate the association between individuals’ levels of concern, knowledge, and behaviors related to COVID-19.

**Methods:**

The questionnaire, which consisted of 15 closed-ended questions, was designed to investigate Chinese netizens’ knowledge about COVID-19. The self-selection online survey method of nonprobability sampling was used to recruit participants through Dingxiangyisheng WeChat (a public, medical, and health service platform in China) accounts. Standard descriptive statistics and multivariate logistic regression analyses were conducted to analyze the data.

**Results:**

In total, 10,304 respondents were surveyed on the internet (response rate=1.75%; 10,304/590,000). Nearly all (n=9803, 95.30%) participants were concerned about “confirmed cases” of COVID-19, and 87.70% (n=9036) received information about the outbreak through social media websites. There were significant differences in participants’ concerns by sex (*P*=.02), age (*P*<.001), educational attainment (*P*=.001), and occupation (*P*<.001). All knowledge questions and preventive practices were associated with concerns about COVID-19. The results of the multivariate logistic regression indicated that participants’ sex, educational attainment, occupation and employment status, knowledge acquisition, and concern level were significantly associated with the practice of proper preventive behaviors.

**Conclusions:**

This study elucidated Chinese netizens’ concerns, information sources, and preventive behaviors related to the COVID-19 pandemic. Sex, educational attainment, occupation and employment status, knowledge acquisition, and level of concern were key factors associated with proper preventive behaviors. This offers a theoretical basis for the government to provide targeted disease prevention and control information to the public.

## Introduction

In December 2019, the first cases of COVID-19 were detected in Wuhan, Hubei Province, China. Soon after, the pandemic spread rapidly across China and abroad [1]. As a result of the rapidly increasing numbers of confirmed cases and deaths, the public paid unprecedented attention to the pandemic [2]. By February 1, 2020, 14,380 cases and 304 deaths had been confirmed in China [3].

Internet access has increased worldwide during the past decade, reaching 48% of the world population in 2017 [4]. As of December 31, 2018, there were 829 million internet users in China (a penetration rate of 59.6%) [5]. Compared with severe acute respiratory syndrome (SARS) in 2003 [6], the Middle Eastern respiratory syndrome (MERS) [7], and the Zika virus [8], people are more willing to learn about the current pandemic and acquire relevant protection knowledge over the internet [9]. As there is currently neither a vaccine nor a specific drug treatment for COVID-19, performing personal protection behaviors is still the most effective measure to control the pandemic.

Local governments have responded with a series of interventions to raise public concerns [10], including raising the level of emergency response [11] and traffic restrictions [12]. Concurrently, disease prevention and control organizations have employed varied means of health education to encourage the public’s preventive behaviors. For example, the Chinese Center for Disease Control and Prevention (China CDC) issued COVID-19 Guidelines for Public Protection (version 2). A printed edition was distributed in the community, and an downloadable electronic version was made available on the official website [13]. Further, the local mainstream media, websites, and a 24-hour telephone hotline disseminated information about behaviors to prevent respiratory communicable diseases. Previous research has found that the more the public pays attention to public health emergencies, the easier it will be for them to take the initiative to seek knowledge regarding disease-related prevention and control, adopt correct personal protective behaviors, and improve support for local disease-related prevention and control policies [14,15]. There is also a large number of scientific literature that reports on people’s knowledge, attitudes, and behaviors related to infectious diseases [6,8]. However, to the best of our knowledge, there is limited research addressing the relationships between the public’s level of concern and knowledge of and behaviors related to infectious disease prevention [16]. Based on the above theory, our hypothesis is that there may be a certain association between the public’s concerns, knowledge, and preventive practices related to COVID-19. In addition, raising the level of public concern regarding the risks related to COVID-19 may be an important prerequisite in influencing the public’s understanding, and may be a key entry point in enhancing self-protection.

Hence, our main objectives were to determine Chinese netizens’ concerns related to COVID-19 and its relationship with internet content, and to elucidate the association between individuals’ concerns, knowledge, and preventive practices related to COVID-19.

## Methods

### Design

Following Facebook and Twitter’s success in medical education in Western universities [17], WeChat is a new media app that is currently recognized as the best social networking site to use for a similar purpose, given that its users span all ages and professions in China [18]. There are currently an estimated 963 million WeChat users in China [5]. As a result, WeChat has been widely utilized in medical fields and has had a positive influence. Dingxiangyisheng WeChat is a public, medical, and health service platform in China. It mainly provides reliable online consultation services, health knowledge lectures, professional science articles, and other services for the domestic public. As the largest professional medical website in China, it has more than 5.5 million users and receives millions of daily visitors. Its users are mainly those who care about their health and that of their families, especially young Chinese mothers.

This study used opportunity sampling. A message with a questionnaire related to COVID-19 was sent to all users with a Dingxiangyisheng WeChat account. Individuals who were users of a WeChat account could participate in this study through their mobile phone or computer. In summary, 590,000 users read this information, and 10,304 of them volunteered to participate in this survey. To avoid repeated questionnaire submission, each respondent could only submit the questionnaire once per registered phone number. Questionnaires could not be revised or repeated after submission. This study was approved by the Ethics Committee of the Zhejiang Provincial CDC.

### Participants

A cross-sectional, population-based internet survey was conducted from January 31 to February 2, 2020, at the beginning of the COVID-19 pandemic in China [19]. Participants were aged ≥15 years and lived in China. Participation was voluntary without any financial incentive. Participants' personal information was kept confidential and stored at Dingxiangyisheng. Participants who did not fully complete the questionnaire were excluded.

### Questionnaire

The online questionnaire, designed by health educational experts from the Zhejiang CDC, consisted of 15 closed-ended questions that aimed to collect the following information from respondents (Multimedia Appendix 1):

1. Sociodemographic and background information, including age, sex, province, occupation and employment status, educational attainment, and infection status (6 questions).
2. Concerns related to COVID-19 (1 question). A 3-point Likert-type scale was used to ascertain the level of concern (1=very, 2=general, and 3=not at all).
3. Participants’ actual knowledge of COVID-19, including four aspects: its incubation period, transmission routes, symptoms, and knowledge of personal disease prevention behaviors (4 questions).
4. Practices of preventive measures against COVID-19, including mask wearing, handwashing with soap at home, avoiding public places and public transportation, and proper way to sneeze (4 questions).

Prior to administering the online survey, the questionnaire was pilot tested using the interception sampling method. Inspectors stopped passersby at a regular intersection and asked if they would be willing to answer the survey, which took approximately 10 minutes to complete. The survey questionnaire was pilot tested in January 2020 (N=100) to ensure practicability and interpretability of answers. For our online survey (N=10,304), the overall Cronbach α value was 0.771. The Kaiser-Meyer-Olkin test coefficient was 0.578. By factor analysis, the cumulative contribution rate of the four factors with a characteristic root greater than 1 was 44.866, indicating that the questionnaire has good reliability and validity.

### Data Analyses

Data were analyzed using SPSS, version 13.0 (IBM Corp). Sociodemographic and background information was converted into classification variables or grade variables, and the corresponding composition ratio was calculated. For questions about knowledge and practices of preventive measures, answers of “yes” were coded into the correct knowledge and practices, while answers of “no” and “unclear” were coded into incorrect knowledge and practices. The nonparametric Mann-Whitney test was used for comparisons among groups with different characteristics, and the Kruskal-Wallis H test was used for comparisons among multiple groups. Questions regarding knowledge and personal protective measures were expressed by dichotomous variables. Mann-Whitney *U* tests of two independent samples were conducted to identify apparent associations between individuals’ concerns with knowledge and practices of preventive measures. A *P* value <.05 was considered significant.

## Results

### Participants’ Characteristics

A total of 10,304 individuals completed the online survey (response rate=1.75%; 10,304/590,000). Participants’ sociodemographic characteristics are shown in Table 1.

**Table 1:** Participants’ sociodemographic characteristics (N=10,304).

Variable		Participants, n (%)
**Sex**		
	Male	2670 (25.91)
	Female	7634 (74.09)
**Age (years)**		
	15-20	901 (8.74)
	21-30	4830 (46.88)
	31-40	2945 (28.58)
	41-50	1141 (11.07)
	51-60	403 (3.91)
	≥60	84 (0.82)
**Educational attainment**		
	No education	92 (0.89)
	Primary	434 (4.21)
	Preparatory	1117 (10.84)
	Secondary	7219 (70.06)
	University graduate	1442 (13.99)
**Occupation and employment status**		
	Government and public institution staff	1729 (16.78)
	Enterprise/commercial/service worker	4100 (39.79)
	Farmer	104 (1.01)
	Retired	202 (1.96)
	Homemaker	751 (7.29)
	Student	1894 (18.38)
	Unemployed	395 (3.83)
	Medical staff	668 (6.48)
	Other	461 (4.47)
**Infection status**		
	Confirmed case	0 (0)
	Suspected case	29 (0.28)
	Close contact with a confirmed case	293 (2.84)
	None of the above	9982 (96.88)

### Participants’ Concerns and Their Information Sources

Table 2 presents data regarding participants’ information sources and what they were concerned about. Nearly all participants were concerned about the number of confirmed cases. Most of the information about the outbreak was received through a social media website, while far less was obtained through traditional mass media (eg, television and newspapers).

**Table 2:** Participants’ concerns and information sources related to COVID-19.

Variable		Participants, n (%)
**Concern**		
	Confirmed cases	9803 (95.30)
	Number of deaths	8463 (82.27)
	Number of recovered	8131 (79.04)
	Suspected cases	6942 (67.48)
	Number of severe patients	4795 (46.61)
**Information source**		
	Social media website	9036 (87.70)
	Smartphone app	4556 (44.22)
	Television	4054 (39.35)
	Friends/relatives	2447 (23.75)
	Government website	2201 (21.36)
	Short message service	1111 (10.78)
	Community outreach	1070 (10.39)
	Newspaper	679 (6.59)
	Significant other/partner	166 (1.61)

### Concerns According to Participants’ Characteristics

Since the number of participants who reported they were “unconcerned” was very small, we merged the “unconcerned” and “general” categories, resulting in two categories for level of concern: “very” concerned or “general/unconcerned.” Table 3 presents the concerns according to participants’ characteristics. There were significant differences in concerns by sex, age, educational attainment, and occupation—specifically, women, those who were older, those who were more educated, and those who were retired expressed a greater level of concern than their counterparts. Students were least concerned compared to other occupation types. There were marginally significant differences between infection status and concerns; those with close contact with a confirmed case were more concerned about COVID-19 than the other types listed in Table 3.

**Table 3:** Concerns about COVID-19 according to participants’ characteristics.

Variable		Very (n=8112), n (%)	General/not at all (n=2177), n (%)	Z or χ^2^	*P* value
**Sex**				χ^2^=2.42	.02
	Male	2146 (80.37)	524 (19.63)		
	Female	5966 (78.15)	1668 (21.85)		
**Age (years)**				Z=318.28	<.001
	15-20	610 (67.70)	291 (32.30)		
	21-30	3542 (73.33)	1288 (26.67)		
	31-40	2517 (85.47)	428 (14.53)		
	41-50	1004 (87.99)	137 (12.01)		
	51-60	364 (90.32)	39 (9.68)		
	≥60	75 (89.29)	9 (9.71)		
**Educational attainment**				Z=11.97	.001
	No education	70 (76.09)	20 (21.91)		
	Primary	340 (78.34)	93 (21.66)		
	Preparatory	846 (75.74)	270 (24.26)		
	Secondary	5678 (78.65)	1530 (21.34)		
	University graduate	1178 (81.69)	264 (18.31)		
**Occupation and employment status**				Z=35.50	<.001
	Government and public institution staff	1461 (84.50)	268 (15.50)		
	Enterprise/commercial/service worker	3234 (78.88)	866 (21.12)		
	Farmer	85 (81.73)	19 (18.27)		
	Retired	181 (89.60)	21 (10.40)		
	Homemaker	613 (81.62)	138 (18.38)		
	Student	1300 (68.64)	594 (31.36)		
	Unemployed	286 (72.41)	109 (27.59)		
	Medical staff	572 (85.63)	96 (14.37)		
	Other	380 (82.43)	81 (17.57)		
**Infection status**				Z=5.83	.054
	Suspected case	18 (62.07)	11 (37.93)		
	Close contact	234 (79.86)	59 (20.14)		
	None of the above	7860 (78.74)	2122 (21.26)		

### Concerns About COVID-19 According to Related Knowledge and Preventive Practices

For all knowledge questions, participants who were more (vs less) concerned about COVID-19 were significantly more likely to master relevant knowledge. For almost all practice-related questions, participants who had more (vs less) concerns about COVID-19 were significantly more likely to take the right action to avoid SARS-CoV-2 infection. Notably, less concerned participants were more likely to avoid public places and public transportation than other participants (Table 4).

**Table 4:** Concerns about COVID-19 according to related knowledge and preventive practices.

Variable		Very, n (%)	General/not at all, n (%)	Total, n (%)	χ^2^	*P* value
**Knowledge items (proportion answered correctly)**						
	Incubation period	7041 (86.80)	1829 (83.51)	8870 (86.08)	16.24	<.001
	Transmission routes	6212 (76.58)	1619 (74.05)	7831 (76.00)	6.99	.009
	Symptoms	6675 (82.29)	1663 (76.21)	8338 (80.92)	46.05	<.001
	Personal preventive knowledge	7020 (86.54)	1826 (83.37)	8846 (85.85)	14.87	<.001
**Preventive practices**						
	Knows how to wear a mask when going out	7916 (97.58)	2111 (96.42)	10,027 (97.31)	10.79	<.001
	Washes hands after getting home	7548 (93.05)	1878 (85.90)	9426 (91.48)	120.32	<.001
	Avoids going to public places and using public transportation	2160 (26.63)	519 (23.61)	2679 (26.00)	7.81	.005
	Can cough or sneeze properly	4822 (59.44)	1142 (52.18)	5964 (57.88)	38.18	<.001

### Multivariate Logistic Regression

For the analysis, respondents who answered the 4 knowledge questions correctly were deemed to have mastered COVID-19 preventive knowledge; otherwise, they were deemed to have not mastered it. Similarly, respondents who correctly answered the 4 questions related to preventive practices were regarded as having correct preventive practices, while those who did not were regarded as not having correct preventive practices. Preventive practices were used as the dependent variable, while sex, age, educational attainment, occupational status, and knowledge acquisition were used as independent variables in the multivariate regression analysis. The multivariate logistic regression revealed the factors that contributed to having correct preventive practices (Table 5). Women were more likely than men (*P*=.001) to practice the correct precautions. Participants with more education (secondary, *P*=.03; university graduate, *P*=.01) were more likely to perform the right preventive practices than those with less educational attainment. Students were less likely to take the right preventive practices compared to other occupational groups (*P*=.02). Concern (*P*<.001) and knowledge acquisition (*P*=.001) regarding the COVID-19 outbreak were significantly associated with proper preventive practices.

**Table 5:** Multivariate logistic regression of factors that contributed to performing preventive practices.

Variables		B	SE	Wald χ^2^	*P* value	Odds ratio	95% CI
**Sex (reference: male)**							
	Female	–0.22	0.07	10.84	.001	0.80	0.70-0.91
**Educational attainment (reference: no education)**							
	Secondary	–0.26	0.12	4.71	.03	0.77	0.61-0.98
	University graduate	–0.20	0.08	6.01	.01	0.82	0.70-0.96
**Occupation and employment status (reference: government and public institution staff)**							
	Student	0.38	0.17	5.19	.02	1.46	1.05-2.02
**Knowledge acquisition (reference: no)**							
	Yes	–0.19	0.06	10.98	.001	0.83	0.74-0.93
**Concern (reference: very)**							
	General/not at all	0.48	0.08	38.16	<.001	1.61	1.39-1.88

## Discussion

### Principal Findings

This study elucidated the concerns and information sources related to the COVID-19 pandemic of Chinese netizens. We also determined the relationship between these individuals’ concerns and their personal protection knowledge and behaviors.

Our results revealed that the vast majority of the sample were very concerned about this outbreak. Among all surveyed participants, 78.73% were “very concerned,” which is significantly higher than that found in a study involving Chinese residents during the SARS pandemic [20]. On the one hand, as the pandemic developed rapidly in early February, the Chinese government and relevant departments took a variety of strict prevention and control measures, including disseminating information through various media platforms [10,12]. On the other hand, with the continuous popularization of the internet in China, the public has more opportunities to obtain information that directly concerns them than ever before, which motivates them to actively take preventive measures [21].

The number of confirmed COVID-19 cases per day was the item that nearly all participants were concerned about. In response, China's National Health Commission and health departments at all levels announce the number of confirmed cases in cities and counties daily through official media websites to keep the public informed of new outbreaks in the region. With the rapid development of the internet worldwide, the way the public obtains health information has changed greatly since the SARS [6], H7N9 [22], and Zika [23] outbreaks. Specifically, the proportion of traditional media, such as television and newspapers, has gradually decreased, while the proportion of new media, such as websites and smartphone apps, has gradually increased [24]. This suggests that, when there is a new infectious disease outbreak, the government should release information to the public through an official website and other relevant media to increase public awareness as soon as possible, and ensure that the correct information is disseminated to foster protective knowledge and behavior.

Consistent with previous studies [25], we found that most people have the right knowledge to prevent COVID-19, especially those who were “very” concerned about the pandemic. Surprisingly, we found that the percentage of respondents electing to avoid going to public places and using public transportation was “very low” compared to other personal preventive practices. It is worth noting that the outbreak happened during the Spring Festival holiday in China; thus, population mobility had increased. Even in the face of such a severe outbreak, most people did not avoid going to public places or using public transportation [12].

As in previous studies [24,26], we found a statistically significant association between sex and personal preventive practices. Women are more likely than men to take the right preventive practices. Although a univariate analysis found significant statistical differences between age and personal preventive practices, multivariate logistic regression analysis did not find such differences. This disparity in the results might be explained by the collinearity between age and educational attainment when we conducted the multivariate logistic regression analysis. Students were significantly less concerned about the outbreak than other occupational groups. Moreover, students were less likely to take proper preventive practices than other occupational groups. Students who were less interested in acquiring information about diseases and utilizing protective measures would increase their risk of infection [8]. Thus, before returning to school, students should be educated about the transmission of COVID-19 and how to develop good personal hygiene habits.

Increased attention to the pandemic was associated with increased knowledge about prevention and control, which coincides with previous results [27]. This shows that knowledge acquisition and the adoption of appropriate behaviors are closely related to individuals’ disease-related concerns. For individuals who are not concerned, stakeholders need to increase health education and ensure they receive the appropriate information.

### Strengths and Limitations

The strength of our study is its large sample, which was recruited during a critical period—the early stages of the COVID-19 outbreak. Similar to the results of a recent online survey about COVID-19 [28], our sample was overrepresentative of women, well-educated people, and white-collar workers.

There are several limitations to this study. First, owing to limited access to the internet and online health information resources, certain populations such as the elderly and rural residents were insufficiently represented; therefore, this study is not representative of all Chinese people. The study, which recruited participants through Dingxiangyisheng WeChat, had a bias in favor of women, especially young Chinese mothers. Second, we employed a cross-sectional design; therefore, we only revealed correlations between variables and cannot infer causal links between them. More in-depth scientific research is needed.

### Conclusions

This study elucidated the concerns, information sources, and preventive behaviors of Chinese netizens related to the COVID-19 pandemic. Sex, educational attainment, occupation and employment status, knowledge acquisition, and concern level were key factors associated with proper preventive behaviors. This offers a theoretical basis for the government to provide targeted disease prevention and control information disseminated to the public.

## Appendix

Multimedia Appendix 1 COVID-19 prevention and treatment questionnaire in Chinese.

## Abbreviations

China CDC: Chinese Center for Disease Control and Prevention
MERS: Middle Eastern respiratory syndrome
SARS: severe acute respiratory syndrome
