# Supplementary material for: Concerns About Information Regarding COVID-19 on the Internet: Cross-Sectional Study
Source: J Med Internet Res. 2020 Nov 9;22(11):e20487. doi: 10.2196/20487 (PMC7655729; doi:10.2196/20487)

新型冠状病毒感染的肺炎防治健康教育调查问卷

今日，我国多地出现新型冠状病毒感染的肺炎病例。为了解大众对相关健康知识的需求，以更好地进行疾病防控工作，我们邀请您参加“新型冠状病毒感染的肺炎防治健康教育调查”。您的个人信息和回答内容将被严格保密。希望您支持我们的工作，谢谢！如果您愿意参加本次调查，请您点击您认为符合您的想法的选项。

1.您的性别：①男 ②女

2.您的年龄：①20岁及以下 ②21～30岁 ③31～40岁 ④41～50岁

⑤51～60岁 ⑥60岁及以上

3.文化程度：①小学及以下 ②初中 ③高中/职高/中专 ④大专及本科 ⑤硕士及以上

4.职业：①政府事业单位工作人员 ②企业/商业/服务业工作人员 ③工人 ④农民 ⑤离退休人员 ⑥家庭主妇 ⑦学生 ⑧无业 ⑨医务人员 ⑩其他

5.您的状态符合以下哪种？

①确诊患者 ②疑似患者 ③密切接触者 ④以上都不是

6.关于新型冠状病毒感染的肺炎疫情，您的关注程度？

①非常关注 ②一般关注 ③不关注

7.您关注哪些疫情信息？

①确诊人数 ②死亡人数 ③治愈/出院人数 ④疑似人数 ⑤重症患者人数

8.您主要通过什么渠道了解新型冠状病毒感染的肺炎的防护知识？[可多选]

①家人/朋友/同事 ②政府网站（如疾控中心） ③重要伙伴 ④电视 ⑤社会媒体网站 ⑥报纸 ⑦手机短信 ⑧社区宣传 ⑨新闻资讯类APP（如今日头条、新浪新闻等）

9.您认为新型冠状病毒感染的肺炎最长潜伏期为多少天？

①2天 ②7天 ③12天 ④14天 ⑤不知道

10.新型冠状病毒主要传播途径有哪些？（可多选）

①飞沫传播 ②接触传播 ③消化道传播 ④血液传播 ⑤不知道

11..据您的了解，新型冠状病毒感染的肺炎主要有哪些表现？

①皮疹 ②乏力 ③腹泻 ④高热 ⑤腹痛

⑥干咳 ⑦流涕 ⑧持续低热 ⑨咳痰 ⑩呼吸困难 ⑪其他 ⑫不知道

12.您认为以下哪些措施能够保护自己和家人，避免感染新型冠状病毒？（可多选）

①呆在家里不外出 ②开窗通风 ③房间熏醋 ④出门戴口罩

⑤勤洗手 ⑥补充维生素C ⑦吃抗生素 ⑧其他

13.您会按以下哪种方式佩戴口罩？

①
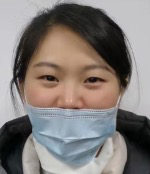
 ②
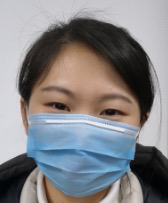
 ③
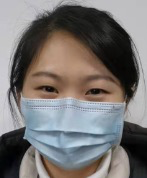
 ④
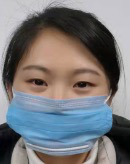
 ⑤不知道

14.在疫情爆发期间，你回家时会用自来水洗手吗?

15.疫情爆发期间，你是否避免去公共场所或使用公共交通工具?

①是 ②否 ③不知道

16.您平时咳嗽或者打喷嚏是下列哪种情形？

①毫无遮掩的在人群中直接打喷嚏或咳嗽
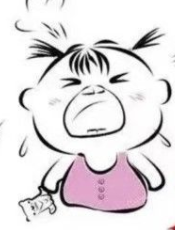


②转头不直面人群的打喷嚏、咳嗽
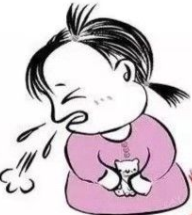


③用手遮掩口鼻的打喷嚏、咳嗽
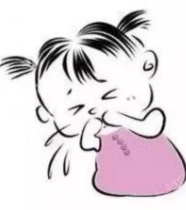


④弯曲肘部遮挡口鼻或使用纸巾捂住口鼻
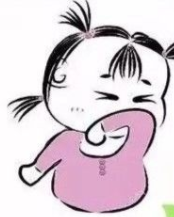

Supplement: Multimedia Appendix 1 [file jmir_v22i11e20487_app1.doc]
